# Role of Extracellular Vesicles on Cancer Lymphangiogenesis and Lymph Node Metastasis

**DOI:** 10.3389/fonc.2021.721785

**Published:** 2021-09-07

**Authors:** Linlin Wang, Ling Li, Guiquan Zhu

**Affiliations:** ^1^Department of Stomatology, Sichuan Cancer Hospital, Sichuan Key Laboratory of Radiation Oncology, School of Medicine, University of Electronic Science and Technology of China, Chengdu, China; ^2^State Key Laboratory of Oral Diseases, National Clinical Research Centre for Oral Diseases, Department of Head and Neck Oncology, West China Hospital of Stomatology, Sichuan University, Chengdu, China

**Keywords:** extracellular vesicles (EVs), exosomes, cancer, lymphangiogenesis, lymph node metastasis (LNM).

## Abstract

Lymph node metastasis (LNM) of tumors is an established indicator of poor prognosis in patients. Tumor-associated lymphangiogenesis is a key step in LNM and has gained much attention. However, currently, there is no anti-tumor lymphangiogenesis drug used in clinical practice. Recently, studies on extracellular vesicles (EVs) have shown that different types of cells in the tumor microenvironment can release EVs that encapsulate a variety of molecules, including proteins, nucleic acids, and metabolites. Lymph endothelial cells (LECs) regulate tumor lymphangiogenesis through the uptake of EVs packed with different biologically active contents. In this review, we will discuss the possible mechanisms by which EVs participate in the regulation of tumor-associated lymphangiogenesis and LNM, summarize the potential value of EVs that can be used as biomarkers for the determination of tumor LNM, and indicate the potential anti-tumor lymphangiogenesis therapy.

## Background

The spread of cancer cells is the main cause of death in patients with solid tumors (1), such as melanoma, head and neck squamous cell carcinoma (HNSCC), and colorectal cancer. Lymph nodes (LNs) are the starting site of cancer cell metastasis from the primary site to distant organs. Studies have shown that cancer cells may spread to sentinel lymph nodes (SLNs) through lymphatic vessels, facilitating distant metastasis of cancer cells (2). Thus, regional LNM is of vital significance for cancer patients, which not only plays a decisive role in the choice of treatment but is also closely related to the poor prognosis of patients (3). In the last decade, much attention has been paid to the molecular mechanism of tumor lymphangiogenesis and LNM, because targeting tumor-associated lymphangiogenesis has been expected to effectively inhibit cancer progression.

Extracellular vesicles (EVs) are nano-sized natural membrane vesicles released by various cell types, with diameters ranging from 30 to 1,000 nm. They carry a variety of biologically active substances from parental cells, including proteins, nucleic acids, metabolites, and lipids. EVs play important roles in cell-to-cell communication by carrying and transmitting different bioactive molecules, which is particularly important for the formation of cancer metastasis (4, 5). Studies have shown that tumor cell-derived EVs (TEVs) can transfer proteins and nucleic acids to target cells to regulate tumor lymphangiogenesis and lymphatic network reconstruction, which promote LNM and distant spread of the tumor (6–8).

## Lymphatic System Promotes Tumor Progression

Tumor-induced lymphangiogenesis plays a significant role in promoting tumor growth and metastasis (2). Tumor-associated lymphatic vessel density is closely correlated with sentinel LNM, distant metastasis, and patient survival (9, 10). The lymphatic system mainly promotes the malignant progression of tumors in three ways: 1) lymphatic vessels pave ways for local and distant metastasis of tumor cells; 2) as a regulator of the immune system, lymphatic vessels transport tumor antigens and immune cells to induce an anti-tumor immune response; or 3) lymphatic vessels can provide a suitable niche for cancer stem cells, maintaining the stemness of stem cell-like tumor cells, and the potential for tumor recurrence. In melanoma, dendritic cells can deliver tumor antigens to draining lymph nodes through the lymphatic vessel, which drives T cell activation (11). Impaired lymphatic vessels reduce the levels of tumor-associated antigens in the draining lymph nodes of model mice and inhibit anti-tumor immunity (12). In addition, LECs can interact with various immune cells to regulate immune cell activity. By the above-mentioned means, lymphatic vessels play important roles in the malignant progression of tumors.

## Mechanism of Lymphangiogenesis

Lymphangiogenesis, also known as lymphangiectasia, is a key step in the metastasis of malignant cells through LNs. This process is regulated by tumor cells and affected by the cytokines secreted by stromal cells and infiltrating inflammatory cells in the tumor microenvironment (TME) (13–16).

### Vascular Endothelial Growth Factor Signaling Pathways

The VEGFC/VEGFD-VEGFR3 axis is the most recognized pathway among the related signaling pathways that regulate lymphangiogenesis (15). A large number of studies have confirmed that the expression levels of VEGFC and VEGFD in many types of tumor cells are correlated with tumor-associated lymphangiogenesis, tumor cell invasion into lymphatic vessels, and LNM (17). The upregulation of VEGFC/D expression can promote tumor-associated lymphangiogenesis and increase tumor metastasis to local LNs and distant organs, which can be inhibited by blocking the binding of VEGFC/D to VEGFR-3 (18–20). In addition to VEGFC/D in the VEGF family, VEGFA has been found to be involved in tumor lymphangiogenesis. A study by Hirakawa et al. showed that primary tumors with VEGFA overexpression induced lymphangiogenesis in SLNs before metastasis to LNs (21).

### Other Signaling Pathways

Increasing evidence has shown that chemokines play important roles in lymphangiogenesis and LNM, in addition to the VEGF family. CCL21/CCR7 and CCL19/CCR7 signaling pathways have gained the most attention because they mediate the homing of immune cells to LN (22). VEGFC can upregulate the expression of CCL21 in lymphatic vessels, driving tumor cells to express CCR7, and enhance the invasive phenotype of tumor cells (23). Other chemokine receptors, such as CXCR2, CXCR3, and CXCR4, have also been shown to contribute to lymphangiogenesis (24–26). ANG-TIE signaling also plays an indispensable role in lymphatic maturation and remodeling. ANG1 and ANG2 contribute to angiogenesis, lymphangiogenesis, and metastasis by binding to TIE1 and TIE2 on vascular endothelial cells, LECs, and pericytes (27, 28).

In addition to the above-mentioned pathways and factors, other factors, such as platelet-derived growth factor-BB, insulin-like growth factors 1 and 2, fibroblast growth factor, prostaglandins, hepatocyte growth factor, sphingosine-1-phosphate, adrenomedullin, and interleukin-7, have also been suggested to be involved in the regulation of tumor lymphangiogenesis (17). Tumor lymphangiogenesis is regulated mainly by the VEGF family and angiopoietins, with many other factors and chemokines acting indirectly by regulating VEGF signaling (28).

## Role of EVs in Tumor Lymphangiogenesis

**Figure 1:**
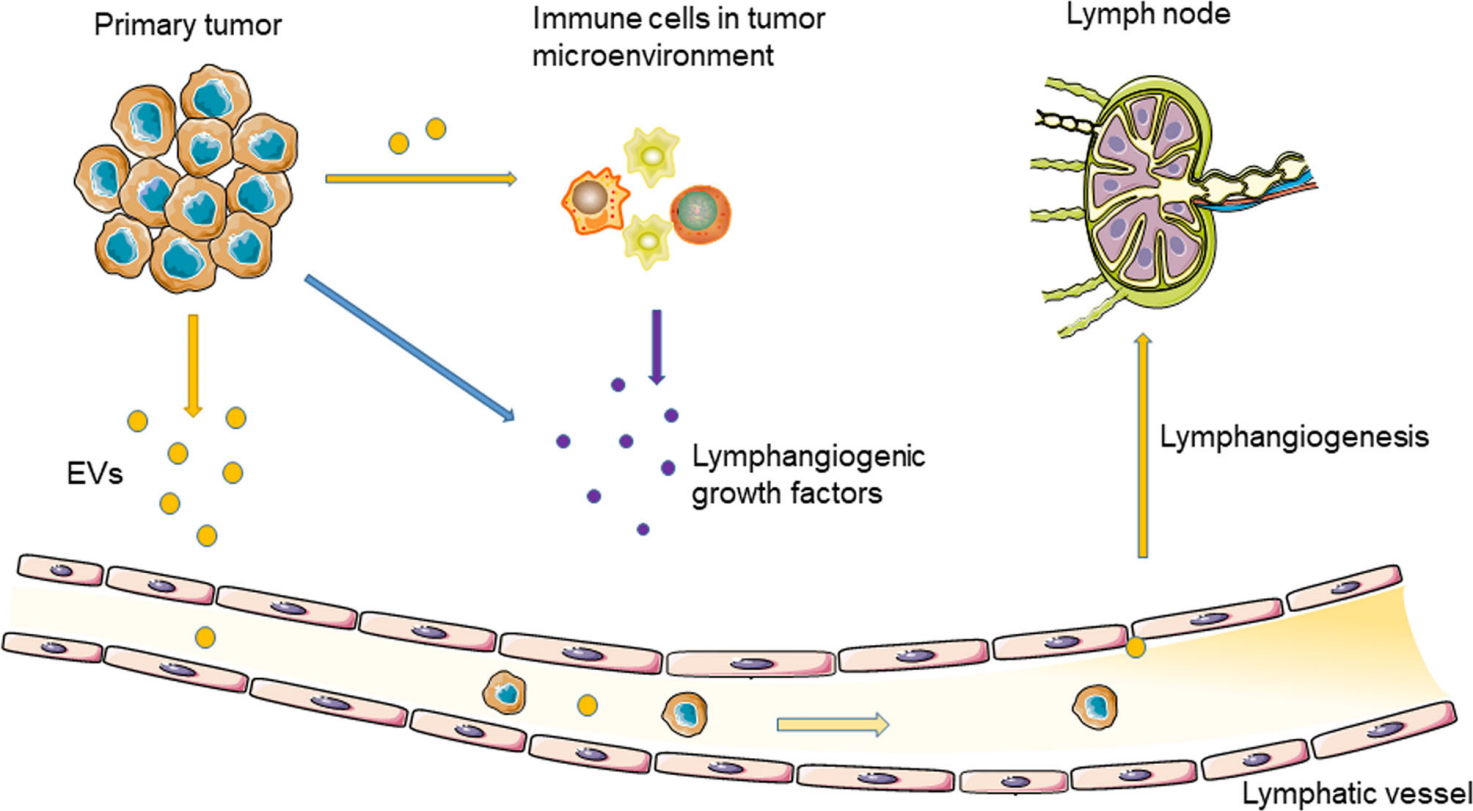
Tumor-derived EVs regulate tumor lymphangiogenesis and lymphatic metastasis through delivering nucleic acids and proteins.

As a natural nanoscale vesicle, EVs can pass through the interstitial matrix entering the lymphatic circulation (29), which makes EVs ideal carriers for message transport between the lymphatic system and tumor cells (30). In addition, due to the unique lipid bilayer structure of EVs, proteins, nucleic acids, and other molecules carried by EVs are protected from degradation in the extracellular environment (29). In 2001, Hood et al. proposed that EVs secreted by tumor cells can reach SLNs to prepare a pre-metastatic niche for tumor metastasis (31). Therefore, TEVs may induce tumor lymphangiogenesis and lymphatic invasion (**Figure 1**).

### EV Characteristics

In the past decade, a booming interest has been paid to EVs in cancer research, mainly because of the discovery of functional molecular cargos in EVs that allow them to operate as signaling platforms for information delivery between cells. As key messengers in intercellular communication, EVs are involved in a variety of physiological and pathological processes, including growth and development, immune regulation, homeostasis of the internal environment, neurodegenerative diseases, and cancer (32). In the early 1960s, Bonucci and Anderson found that chondrocytes can release small vesicles with a diameter of 100 nm (33, 34). At the same time, Wolf demonstrated that platelets could release small vesicles (called “platelet dust”), which had a clotting function similar to that of platelets (35). The term “exosome” was first described in reticulocytes during the maturation of erythrocytes by Rose M. Johnstone et al. in the 1980s (36). For a long time, exosomes have been regarded as a nonfunctional metabolic waste excreted by cells, until Raposo discovered in 1996 that exosomes derived from B lymphocytes induce antigen-specific MHC-II-restricted T cell responses, suggesting an active function of exosomes in antigen presentation (37). Thereafter, there has been encouraging progress in exosome research on many aspects of exosome biology, such as biogenesis and release, morphology, contents, isolation technique, and functions.

In addition to exosomes, many other EVs have been identified based on vesicle size, biogenesis, and molecular signature, such as microvesicles and apoptotic bodies. However, most of the literature has disregarded the different origins of exosomes, microvesicles, and other types of nanovesicles, which makes it impossible to identify which ones actually count. Since consensus has not yet emerged on specific markers of EV subtypes, such as endosome-origin “exosomes” and plasma membrane-derived “exosomes” (microparticles/microvesicles), assigning an EV to a particular biogenesis pathway remains extraordinarily difficult (38). Thus, the International Society for Extracellular Vesicles (ISEV) endorses “extracellular vesicle” as the generic term for particles naturally released from the cell that are delimited by a lipid bilayer and cannot replicate (38). In this review, the term extracellular vesicle (EV) was used generally, and exosomes and microvesicles were used when necessary or per reference indicated.

EVs carry a large number of bioactive substances, including proteins, nucleic acids, lipids, and metabolites. The composition and content of EV cargos depend on the type of parent cell and vary between different microenvironments in which the parent cells live. Several databases [i.e., ExoCarta (39), EVpedia (40), and Vesiclepedia (41)] have been built to provide information about EV cargo. Tumor cells and stromal cells utilize EVs to influence the surrounding cells within the microenvironment by transferring RNA and proteins (42). For example, EV-packed RNA plays an important role in cell proliferation, drug resistance, angiogenesis, immunomodulation, and pre-metastasis niche formation, which contribute to cancer progression (43). Additionally, TEVs carry a variety of proteins (i.e., epidermal growth factor receptor variant III, p-glycoproteins, natural killer group 2D (NKG2D) ligands), and transfer these components to receptor cells to promote the propagation of malignancy, drug resistance, and evade anti-cancer immune responses (43). EV proteins and nucleic acids have been intensively studied and have been shown to play important roles in cancer progression. Thus, we will mainly discuss the role of EV proteins and nucleic acids in tumor lymphangiogenesis in this mini review. The updates on how EV metabolites are implicated in cancer progression have been discussed in a recent review (42).

### EVs and Pre-Metastatic Niche

Tumor cell-secreted cytokines, growth factors, and EVs can reshape the extracellular matrix, regulate and reprogram the microenvironment of LNs and other distant organs, and provide a suitable niche for the spread of tumor cells (44–46). TEVs are considered to inhibit innate immune responses by mobilizing bone marrow mesenchymal stem cells and activating tumor-related macrophages and neutrophils (47). Indeed, TEVs produced by different tumor cells have varied metastatic potential and effects on the formation of the pre-metastatic niche. Michael et&nbsp;al. reported that exosomes secreted by non-metastatic melanoma cells activate the immune response by stimulating the expansion of Ly6C^low^ patrolling monocytes (PMo) in the bone marrow, which result in the recruitment of natural killer cells and TRAIL-dependent killing of tumor cells (47). The mechanisms and effects of exosomes on tumor organotropic metastasis are thought to depend on integrin. Specific exosomal integrins mediate the uptake of exosomes by resident matrix cells of specific target organs; for example, exosomal integrins α6β4 and α6β1 are related to lung metastasis, while exosomal integrin αvβ5 is associated with liver metastasis (48). It has been demonstrated that the migration inhibitory factor derived from TEVs mediates the formation of the liver pre-metastatic niche of pancreatic ductal adenocarcinomas (PDAC). TGFβ-promoted fibronectin deposition and recruitment of bone marrow-derived macrophages were suggested to contribute to this process (49).

The formation of the pre-metastatic niche increases angiogenesis and vascular permeability, which contribute to tumor cell dissemination and colonization of specific organs (50). Exosomes produced by melanoma can be taken up by lung endothelial cells, which induce vascular leakage of the pre-metastatic niche and educate bone marrow progenitor cells *via* the MET receptor (51). In melanoma, TEVs have been found to bind to the subcapsular sinus CD169+ macrophages in the drain lymph nodes. When the CD169+ macrophage layer is destroyed, the melanoma exosomes may enter the LN cortex and interact with B lymphocytes, thereby activating B cell immunity (52).

### The Role of EVs in Tumor Lymphangiogenesis

#### EV Proteins

As a key component of EV, proteins play an important role in intercellular communication. Wang et al. found that laminin 332 was significantly upregulated in exocrine bodies isolated from oral squamous cell carcinoma (OSCC) patients with positive LNM compared to healthy people and patients without LNM (6). Laminin 332 knockdown inhibited EV-mediated LEC migration, lymphangiogenesis, and lymph node drainage in LN1-1 cells. After knocking down integrin &alpha;3, the role of laminin γ2-enriched EVs disappeared with lymphangiogenesis, suggesting that the uptake of EVs by LECs is dependent on integrin (6). Moreover, increased expression of interferon regulatory factor 2 (IRF-2) was detected in plasma EVs of colorectal cancer patients with LNM. IRF-2-enriched EVs are ingested by F4/80^+^ macrophages, inducing the release of VEGFC to promote sentinel LNM, LEC proliferation, lymphangiogenesis, and lymphatic network remodeling in LNs (8). Moreover, in a gastric cancer mouse model, Liu et al. confirmed that CD97-enriched EVs promoted LNM of cancer cells (53). Additionally, EVs can promote tumor lymphangiogenesis by intercellularly transferring important lymphangiogenic factors, VEGFC and chemokines. For example, loss of dual-specificity phosphatase-2 (DUSP2) in PDAC promotes the production of the mature form of EV-VEGFC and increases the ability of EVs to carry and transmit VEGFC to LECs, which leads to tumor lymphangiogenesis and tumor cell invasion into lymphatic vessels and promotes early metastasis of pancreatic cancer (54). The role of VEGFC transport by TEVs in regulating the lymphangiogenesis ability of LECs has been well-established (55).

Thus, EVs, as key messengers in cell-to-cell communication, can carry abnormally expressed proteins from parental tumor cells and deliver these proteins to LECs, thereby regulating tumor lymphangiogenesis and LNM.

#### EV Nucleic Acids

The miRNAs and lncRNAs encapsulated in TEVs are recently discovered mediators of cell crosstalk in the TME (7). Several lncRNAs and miRNAs have been shown to be involved in the regulation of lymphangiogenesis and LNM in a variety of cancer types (56–61). Hepatocellular carcinoma (HCC)-associated long noncoding RNA (HANR) promotes lymphangiogenesis of HCC by secreting miR-296-enriched EVs and regulating EAG1/VEGFA signaling in human skin LECs (62). Bladder cancer cell-derived EVs can deliver the lncRNA LNMAT2, which promotes lymphangiogenesis and LNM by recruiting hnRNPA2B1 and increasing the H3K4 trimethylation level in the prospero homeobox 1 (PROX1) promoter (63). In addition, miR-221-3p enriched in cervical squamous cell carcinoma (CSCC)-derived EVs can be transferred to LECs, which promotes lymphangiogenesis and LNM by inhibiting the expression of vasohibin-1 (VASH1) (7). Subsequently, Zhou et al. also demonstrated that miR-142-5p is significantly increased in CSCC-derived EVs, which leads to the downregulation of lymphatic AT-rich interactive domain-containing protein 2 (ARID2) expression and decreased recruitment of DNA methyltransferase 1 (DNMT1) to the interferon-gamma (IFN-γ) promoter. This results in an increase in indoleamine-2Q-3 IFN-γ dioxygenase (IDO) activity in tumor-related lymphatic vessels, which inhibits the anti-tumor immune therapy response (64). EV miRNAs not only regulate cancer-related lymphangiogenesis but also mediate lymphangiogenesis in inflammatory bowel disease. It has been shown that the EVs secreted by VEGFC-treated adipose stem cells contain increased levels of miR-132, which, upon internalization by LECs, promotes the proliferation, migration, and lymphangiogenesis of LECs by directly targeting Smad-7 and activating TGF-β/Samd signaling (65).

Tremendous progress has been made in research on EV-mediated crosstalk between cells within the TME in the last decade. However, the role of EVs in tumor lymphangiogenesis is yet to be clarified. The EV proteins and miRNAs have been demonstrated to participate in the process of tumor lymphangiogenesis and LNM in several cancer types (**Table 1**). These molecules need to be validated in more cancer types and under different circumstances. Moreover, much effort is needed to further clarify the characteristics of EVs that are found to have lymphangiogenesis function, such as size (e.g., small, medium, or large), density (e.g., low, middle, or high), biochemical composition (e.g., VEGFC+ EVs), conditions (e.g., hypoxia), and cell of origin (e.g., squamous cell carcinoma).

**Table 1:** The role of EVs in tumor induced lymphangiogenesis.

Source of EV	Type of EV	Cargos by EV	Mechanism of EVs cargo in cancer lymphangiogenesis	Reference
Colorectal cancer cells	Exosome	IRF-2	induces VEGFC expression in macrophages	(8)
Pancreatic ductal adenocarcinoma cells	EV	VEGF-C	DUSP2 downregulation increases the amount and moving ability of EV-carried VEGFC	(54)
Hepatocellular carcinoma cells	Exosome	miR-296	regulates EAG1 expression and VEGF signaling in HDLEC cells.	(62)
Gastric cancer cells	Exosome	CD97	aided by the soluble fraction	(53)
Oral squamous cell carcinoma cells	EV	Laminin γ2	regulates integrin-α3-dependent uptake of EV	(6)
Various cancer cells	EV	Podoplanin	regulate EVs biogenesis or secretion	(66)
Hepatocarcinoma cells	Exosome	CXCR4	through SDF-1α/CXCR4 axis mediated MMP-9, MMP-2 and VEGF-C secretions	(67)
Bladder cancer cells	Exosome	LNMAT2	recruitment of hnRNPA2B1 and increasing the H3K4 trimethylation level in the PROX1 promoter	(63)
Cervical squamouscell cell carcinoma	Exosome	miR-142-5p	induces IDO expression through ARID2–DNMT1–IFN-γ signaling to suppress and exhaust CD8+T cells.	(64)
Cervical squamous cell carcinoma	Exosome	miR-221-3p	regulates miR-221-3p-VASH1 axis	(7)
Cervical cancer cells	Exosome	miR-1468-5p	promotes lymphatic PD-L1 upregulation and lymphangiogenesis to impair T cell immunity	(68)
Adipose-Derived Stem cells	Exosome	miR-132	targets Smad-7 and regulates TGF-β/Smad signaling	(65)
Bladder cancer cells	Exosome	BCYRN1	upregulates WNT5A expression, which activated Wnt/β-catenin signaling to facilitate the secretion of VEGF-C	(69)
Esophageal cancer cells	Exosome	ultraconserved RNA 189	targets the EPHA2 of HLECs to activates the P38MAPK/VEGF-C pathway in HLECs	(70)

## EVs as Biomarker of Cancer Lymph Node Metastasis

EVs are widely distributed and can be detected in a variety of body fluids, including plasma, urine, tears, saliva, and lymphatic fluids. EV cargos are expected to become important non-invasive biomarkers for early diagnosis and prognosis evaluation of cancer, especially circulating EV miRNAs and lncRNAs, because these molecules can be protected by the lipid bilayer structure from degradation (29). For example, in patients with OSCC, the levels of laminin-332 in plasma EVs in patients with LNM were significantly higher than those in patients without LNM, indicating that laminin-332 carried by EVs could be used to detect OSCC lymph node metastasis (6). Moreover, EV hsa_circRNA_0056616 was detected in lung adenocarcinoma plasma at significantly higher levels than in the corresponding control, suggesting that EV hsa_circRNA_0056616 could be used as a potential tool for predicting LNM of lung adenocarcinoma (71). In patients with thyroid cancer, plasma EV miR-146b-5p and miR-222-3p were both significantly upregulated in patients with LNM (72). In addition, circulating EV PD-L1 and miR-21 are correlated with LNM in HNSCC (73, 74). However, there are many issues that need to be addressed before the clinical application of EVs as cancer biomarkers, for example, the establishment of efficient, reliable, and robust EV isolation techniques.

## Anti-Lymphangiogenesis Therapy

Since lymphangiogenesis is a key step in tumor LNM, targeting tumor-related lymphangiogenesis is considered an effective therapeutic strategy to inhibit LNM. Some receptor tyrosine kinase inhibitors, such as gefitinib, afatinib, and anlotinib, can target the VEGFC-VEGFR3 signaling pathway, which potentially inhibits tumor-related lymphangiogenesis, lymphatic metastasis, and distant organ metastasis (75, 76). However, because these receptor tyrosine kinase inhibitors target multiple tyrosine kinase receptors, it is difficult to identify which molecules and pathways mediate lymphangiogenesis. Since EVs play important roles in tumorigenesis and progression, targeting EVs is emerging as an attractive strategy for cancer therapy, which has been tested in several animal models. For example, engineered exosomes overexpressing miR-92b-3p have strong anti-angiogenic and anti-tumor capabilities in ovarian cancer models (77). This finding provides a new effective strategy for the application of EVs in anti-angiogenic and antitumor treatments. EVs from VEGFC-treated adipose stem cells promoted LEC proliferation, migration, and tube formation, whereas pretreatment with miR-132 inhibitor attenuated VEGFC-dependent lymphangiogenic response (65). Moreover, Wang et al. demonstrated that uptake of laminin γ2-enriched EVs by LECs enhanced *in vitro* lymphangiogenesis, and laminin γ2 knockdown and neutralization impaired EV-mediated LEC migration, tube formation, and uptake by LECs (6). Nevertheless, EV targeting (on EV molecules, EV production, and EV internalization) represent a novel means of anti-tumor lymphatic therapy, which of course needs more efforts to further clarify and validate.

## Concluding Remarks

As an important messenger of intercellular communication, EVs have been known to mediate tumor progression. When detached from parent cells, EVs can transmit biological information from the parent cells to the recipient cells, reprogram the TME, and mediate tumor progression. Current research on EV-mediated tumor lymphangiogenesis have identified a set of EV miRNAs, lncRNAs, and proteins (both tumor cell-derived and stromal cell-derived) that can regulate LEC proliferation and function, as well as tumor lymphangiogenesis and LNM. These initial findings shed light on developing EV-based cancer biomarkers for LNM, as well as on the potential therapeutic strategies for EV-based anti-lymphangiogenesis. Further investigation is warranted to address the direct regulation by EVs on tumor lymphangiogenesis and LNM, and to solve key bottlenecks, such as the standardization of isolation techniques, internal controls, and clinical-grade production, in EV research.

## Author Contributions

All authors listed have made a substantial, direct, and intellectual contribution to the work, and approved it for publication.

## Funding

This work was supported by the National Natural Science Foundation of China (grant No. 81872196, 81672690, 81772900, and 81972541).

## Conflict of Interest

The authors declare that the research was conducted in the absence of any commercial or financial relationships that could be construed as a potential conflict of interest.

## Publisher’s Note

All claims expressed in this article are solely those of the authors and do not necessarily represent those of their affiliated organizations, or those of the publisher, the editors and the reviewers. Any product that may be evaluated in this article, or claim that may be made by its manufacturer, is not guaranteed or endorsed by the publisher.
